# Genomic resources for a commercial flatfish, the Senegalese sole (*Solea senegalensis*): EST sequencing, oligo microarray design, and development of the Soleamold bioinformatic platform

**DOI:** 10.1186/1471-2164-9-508

**Published:** 2008-10-30

**Authors:** Joan Cerdà, Jaume Mercadé, Juan José Lozano, Manuel Manchado, Angèle Tingaud-Sequeira, Antonio Astola, Carlos Infante, Silke Halm, Jordi Viñas, Barbara Castellana, Esther Asensio, Pedro Cañavate, Gonzalo Martínez-Rodríguez, Francesc Piferrer, Josep V Planas, Francesc Prat, Manuel Yúfera, Olga Durany, Francesc Subirada, Elisabet Rosell, Tamara Maes

**Affiliations:** 1 Laboratory of the Institut de Recerca i Tecnologia Agroalimentàries (IRTA)-Institut de Ciències del Mar, Consejo Superior de Investigaciones Científicas (CSIC), 08003 Barcelona, Spain; 2 Oryzon Genomics, Scientific Parc University of Barcelona, 08028 Barcelona, Spain; 3 CIBERehd, Hospital Clínic, 08036 Barcelona, Spain; 4 IFAPA Centro "El Toruño", Junta de Andalucía, 11500 El Puerto de Santa María, Cádiz, Spain; 5 Instituto de Ciencias Marinas de Andalucía, CSIC, 11510 Puerto Real, Cadiz, Spain; 6 Institut de Ciències del Mar, CSIC, 08003 Barcelona, Spain; 7 Department of Physiology, Faculty of Biology, University of Barcelona, 08028 Barcelona, Spain

## Abstract

**Background:**

The Senegalese sole, *Solea senegalensis*, is a highly prized flatfish of growing commercial interest for aquaculture in Southern Europe. However, despite the industrial production of Senegalese sole being hampered primarily by lack of information on the physiological mechanisms involved in reproduction, growth and immunity, very limited genomic information is available on this species.

**Results:**

Sequencing of a *S. senegalensis *multi-tissue normalized cDNA library, from adult tissues (brain, stomach, intestine, liver, ovary, and testis), larval stages (pre-metamorphosis, metamorphosis), juvenile stages (post-metamorphosis, abnormal fish), and undifferentiated gonads, generated 10,185 expressed sequence tags (ESTs). Clones were sequenced from the 3'-end to identify isoform specific sequences. Assembly of the entire EST collection into contigs gave 5,208 unique sequences of which 1,769 (34%) had matches in GenBank, thus showing a low level of redundancy. The sequence of the 5,208 unigenes was used to design and validate an oligonucleotide microarray representing 5,087 unique Senegalese sole transcripts. Finally, a novel interactive bioinformatic platform, Soleamold, was developed for the Senegalese sole EST collection as well as microarray and ISH data.

**Conclusion:**

New genomic resources have been developed for *S. senegalensis*, an economically important fish in aquaculture, which include a collection of expressed genes, an oligonucleotide microarray, and a publicly available bioinformatic platform that can be used to study gene expression in this species. These resources will help elucidate transcriptional regulation in wild and captive Senegalese sole for optimization of its production under intensive culture conditions.

## Background

In recent years, genomic research employing laboratory fish models, such as zebrafish (*Danio rerio*), medaka (*Oryzias latipes*) and pufferfish (*Tetraodon nigroviridis *and *Takifugu rubripes*), has contributed to understanding the mechanisms of vertebrate development and has shed light on poorly understood evolutionary phenomena such as genome duplication and duplicate gene silencing [1]. The use of genomic technologies on fish is also essential to improve aquaculture research for the mass production of target species, which may have a profound impact on human welfare since fish is a major food source for humans. These studies may be very useful in providing a physiological and genetic basis for marker-assisted selection of strains with enhanced growth or disease resistance, or for identifying key genes and genetic networks involved in the production of viable gametes.

Salmonids are important commercial fish and also unique models for evolutionary studies as they are recent tetraploids [2]. A large expressed sequence tag (EST) database and several cross-species and species-specific cDNA microarrays have recently been developed [3-8]. However, although efforts have been made to partially sequence the transcriptome of specific tissues in some species [9-15], there are still few genomic tools available for commercially relevant marine fish, particularly for functional genomics studies.

Flatfish, members of the order Pleuronectiformes, are a relatively large group of teleosts with about 570 extant species [16]. They are benthic and carnivorous, and most are marine species, although about four probably occur only in freshwater. These fish undergo a unique development process, known as metamorphosis, during which one eye migrates across the top of the skull to lie adjacent to the other eye on the opposite side, while the body flattens and lies on the eyeless side [17]. This unique developmental event involves drastic morphological and physiological changes, although its molecular regulation is still poorly understood [18,19]. Flatfish have long been a choice food, with many members of the group (e.g., halibut, flounder, sole, turbot, plaice) being important for commercial fisheries. With the general worldwide decline in wild fishery [20], with an essentially stable, wild catch of flatfish, research into producing them in aquaculture have been underway for the last fifteen to twenty years. Aquaculture of Japanese flounder (*Paralichthys olivaceus*), turbot (*Psetta maxima*), Atlantic halibut (*Hippoglossus hippoglossus*) and others have been successfully achieved, although improvements in the efficiency of the industrial production of most of these species are still needed.

The Senegalese sole, *Solea senegalensis *(Kaup, 1858), is a highly prized flatfish of which intensive culture has increased over the last decade, particularly in Southern Europe [21,22]. However, partly due to the lack of knowledge of the physiological and genetic mechanisms involved, the development of aquaculture is impaired by the lack of methods to control reproduction in captivity and those to improve larval growth and disease resistance. The Pleurogene™ project, an initiative funded by Genome Spain and Genome Canada, was set up to improve understanding of physiological and evolutionary processes influencing the reproduction and larval development and survival of both Senegalese sole and Atlantic halibut in natural environments and aquaculture [23]. This project has developed genomic and proteomic research tools to help achieve these goals [12,24]. Here we report the establishment of an EST database for Senegalese sole containing 5,208 cDNA sequences, and the construction and validation of an oligo-based microarray for the detection of 5,087 putative transcripts from this species. In addition, an interactive bioinformatic platform termed Soleamold, developed to accommodate the EST database and results from microarray and in situ hybridization (ISH) experiments, is presented. Currently, this platform only contains data on Senegalese sole gonad development but it could be extended in the future with data from other tissues and organs, as well as from other flatfish, to become a powerful tool for flatfish genomic research.

## Results and discussion

### EST survey

**Figure 1:**
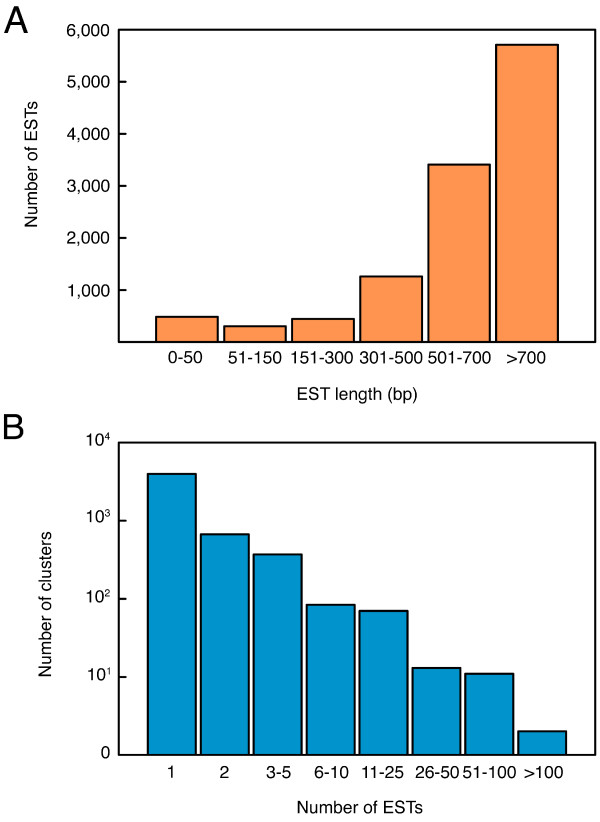
**Distribution of Senegalese sole expressed sequence tags (ESTs) in the normalized multi-tissue cDNA library**. (A) Sequencing read length of ESTs. Read lengths were binned in 100–200 bp increments. Most of the ESTs were more than 700 bp in length. (B) Number of ESTs in each cluster.

Ten non-normalized, directionally-cloned (5' *Eco*RI, 3' *Xho*I) cDNA libraries were constructed from different adult tissues and larval stages (Table 1). For all libraries, the percentage of clones with inserts (recombinant efficiency) was high (97–98%) with average insert sizes between 0.9–1.7 kb. To minimise redundancy before sequencing, aliquots of each library, depending on the titre after one round of amplification (Table 1), were pooled into a master library, which was normalized through three rounds of hybridization. About 10,400 clones from this master library were sequenced with T7 primer for sequences corresponding to the 3' UTR in correctly oriented inserts. Because the general low conservation in 3' UTRs, and genome duplication events that have occurred in teleosts [2], we focused on 3' sequencing to design specific oligos for a microarray to distinguish between potential paralogues arising from gene duplications. After trimming and vector and contaminant removal, 10,185 high-quality sequences were obtained with an average read length of approximately 600–700 bp, with the majority of reads being > 700 bp (Figure 1A). These sequences have been submitted to GenBank (accession numbers: FF281814-FF291996).

**Table 1:** Statistics of *Solea senegalensis *cDNA libraries

Tissue (library)	Recombination efficiency ^a^	Titre (pfu/ml) ^b^	Average insert (kb)	% Representation in master library
**Larvae/Juvenile**				
Premetamorphosis	97.0	4.2 × 10^9^	1.0	1.27
Metamorphosis and postmetamorphosis	97.0	4.0 × 10^9^	0.9	1.93
Abnormal stages ^c^	97.0	2.5 × 10^9^	1.0	2.38
Undifferentiated gonads ^d^	98.4	1.8 × 10^8^	1.4	46.10
				
**Adult**				
Brain (including pituitary gland)	97.3	3.0 × 10^9^	1.2	5.35
Stomach	97.0	6.7 × 10^9^	1.2	0.97
Intestine	97.0	4.1 × 10^9^	1.0	1.88
Liver	97.0	6.6 × 10^9^	1.1	1.17
Ovary ^e^	98.2	2.0 × 10^8^	1.0	32.68
Testis ^f^	97.0	0.5 × 10^9^	1.7	6.26

^a^The recombination efficiency represents the percentage of clones containing inserts; ^b^Titre after one round of amplification; ^c^Larvae showing developmental alterations: albinism, malpigmentation, osteological malformations, and incomplete eye migration; ^d^Three different primary libraries were pooled before amplification; ^e^Mixed tissue from ovaries at pre-vitellogenesis, vitellogenesis and maturation stages; ^f^Mixed tissue from testes at mid and late spermatogenesis, and at functional maturation stage (i.e., spermiating).

The EST sequences were assembled using PHRAP under high stringency to identify clusters (contiguous sequences or contigs) representing redundant transcripts. Clustering analysis yielded 5,208 unique sequences, indicating a redundancy factor of 1.9 (Figure 1B). This compares positively with that obtained in other normalized cDNA libraries available in the UniGene database [25] in which at least 5,000 clones were sequenced from libraries prepared from a mixture of different tissues (e.g., [5,26]). Lower redundancy could possibly be obtained by applying the duplex-specific nuclease method to the cDNA [27], prior to cloning and library generation, which requires mixing all initial RNA samples. However, this method uses a PCR amplification step which may introduce bias against long transcripts, thus selecting for partial (shorter) transcripts. For the libraries to contain as many expressed full-length clones as possible, we consider our normalized library a good compromise between large clone size and efficient normalization.

The most abundant ESTs (represented 30 times or more) in the sole normalized library corresponded to cytoskeletal proteins, vitellogenin Aa and Ab, apolipoprotein A1, zona pellucida (ZP) proteins, elongation factors, creatine kinase 1, lectin, ribosomal proteins and heat shock protein 70 (Table 2). The high abundance of transcripts encoding cytoskeletal proteins, creatine kinase 1, ribosomal proteins and elongation factors may be a consequence of the enrichment of cDNAs of larval origin in the mixed library (30%) since there is a complex reorganization of somatic tissues in sole larvae at metamorphosis and post-metamorphosis [28-32]. Different isoforms of vitellogenins and ZP proteins are known to be highly expressed in the liver of fish females at ovarian recrudescence [33,34], and some ZPs are also highly expressed in the ovary [5,34-36]. Therefore, high levels of vitellogenin and ZP ESTs is consistent with the fact that sexually maturing females were used for the construction of the primary libraries. The over-representation of apolipoprotein A1 and lectin, also observed in cDNA libraries from liver, ovarian follicles and larvae in other fish [12,15,36], may also be related to the physiological stage of the females. Finally, the low but significant abundance of ESTs encoding the stress-related heat shock cognate protein 70 probably reflects the widespread expression of this gene in both larval and adult tissues [37], although it may also suggest a degree of stress caused by the capture and confinement of fish prior to sampling (e.g. [38,39]).

**Table 2:** Largest EST clusters (with ≥ 30 ESTs) in the normalized multi-tissue cDNA library of Senegalese sole

GenBank hit acc. no ^a^	Gene identification (species) of top BLAST hit ^a^	BLAST E-value ^a^	Length (% identity) ^b^	No. ESTs
ABQ58114	Vitellogenin Ab (*Hippoglossus hippoglossus*)	1E-109	185 (78%),	150
CAC45059	Type I keratin S8 (*Oncorhynchus mykiss*)	1E-137	299 (83%)	136
ABQ58113	Vitellogenin Aa (*Hippoglossus hippoglossus*)	0	1293 (73%)	112
CAH59609	Apolipoprotein AI precursor (*Platichthys flesus*)	2E-70	260 (52%)	98
P68140	Actin, alpha skeletal muscle A (*Takifugu rubripes*)	0	377 (100%)	94
BAB12571	Myosin heavy chain (*Pennahia argentata*)	0	779 (95%)	82
AAY21007	Zona pellucida protein Bb (*Sparus aurata*)	3E-139	353 (77%)	78
BAA85157	Elongation factor 1 alpha (*Seriola quinqueradiata*)	0	448 (95%)	64
AAY21009	Zona pellucida protein Ba (*Sparus aurata*)	5E-164	333 (68%)	54
NP_956752	Eukaryotic translation elongation factor 2, like (*Danio rerio*)	0	738 (86%)	52
ABN80443	Creatine kinase 1 (*Poecilia reticulata*)	0	359 (94%)	47
P61155	40S ribosomal protein S19 (*Pagrus major*)	8E-64	121 (95%)	46
NP_571231	Keratin 5 (*Danio rerio*)	2E-138	270 (75%)	44
P84335	Tropomyosin (*Pennahia argentata*)	6E-139	279 (98%)	44
AAO43607	Serum lectin isoform 3 precursor (*Salmo salar*)	7E-17	45 (34%)	39
CAA63709	Chorion protein (*Sparus aurata*)	2E-149	283 (63%)	37
ABB17040	Heat shock cognate 70 (*Fundulus heteroclitus*)	3E-100	198 (96%)	32
BAA95131	Myosin light chain 2 (*Pennahia argentata*)	2E-87	162 (98%)	30

^a^Most significant BLASTX hit is reported; ^b^Extent of BLASTX hit aligned region (in amino acids), and percent identity over the aligned region.

### EST annotation and gene ontology

The BLAST2GO program uses BLAST to find homologous sequences for input sequences and extracts gene ontology (GO) terms to each hit using existing annotations. These GO terms are assigned to the query sequence to give an assessment of the biological process, the molecular function and the cellular compartments represented. In our case, 3,086 sequences out of 5,208 ESTs (59.2%) did not show significant similarity to any known protein after BLASTX, while providing product or gene names for 1,769 sequences (33.9%) and unassigned protein matches (hypothetical proteins) for 353 sequences (6.7%). The number of identified ESTs in sole was lower when compared with EST projects carried out on other fish species. This could be related to sequencing from the 3'end, with most of the nucleotide sequences corresponding to the 3' UTR, and artifacts produced during cDNA synthesis and cloning.

**Figure 2:**
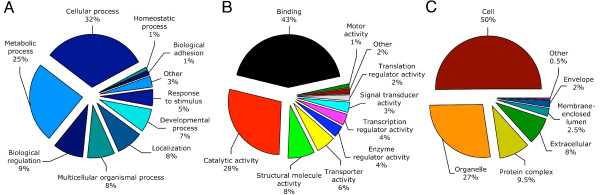
**BLAST2GO categories of Senegalese sole ESTs**. ESTs were analyzed using the BLAST2GO software. Level 2 categories are shown for biological processes (A), molecular function (B), and cellular component (C).

The majority of the functionally annotated sequences from sole had GO assignments for biological process (1,526 ESTs), molecular function (1,613 ESTs) and cellular component (1,395 ESTs) categories (Figure 2). Sequences with GO terms corresponding to biological process fell into 10 categories, with most of the ESTs being dedicated to cellular and metabolic processes (57%), and lower, similar amounts dedicated to biological regulation (9%), multicellular organism processes (8%), localization (8%) and developmental processes (7%) (Figure 2A). Regarding molecular function in the GO resource, defined as 'what a gene product does at the biochemical level', we found that 71% of the ESTs were dedicated to binding and catalytic functions, while the remaining ESTs were mostly dedicated to structural molecule activity (8%) and transporter activity (6%) (Figure 2B). Most of the binding functions were at the intracellular level rather than external and included nucleotide and nucleic acid binding, protein binding and ion binding. Catalytic activities included transferase, hydrolase and oxidoreductase activities. In relation to cellular components, Senegalese sole gene products were generally found associated with the intracellular space (50%) or in organelles (27%), with more associated with membrane bound organelles such as mitochondria (Figure 2C). Almost 9.5% were found in protein complexes, such as the ribonucleoprotein complex.

BLASTX and GO analyses, however, did not give full information on the tissue-specific transcription profiles as the ESTs were derived from a multi-tissue cDNA library. Future sequencing of ESTs in a tissue-specific fashion is necessary for this purpose. Nevertheless, the analyses performed did give information on the nature of the ESTs in the current Senegalese sole database, and identified a number of mRNAs of potential interest during gametogenesis and larval development (Table 3). For some of the ESTs, sequencing information from the 3' end allowed to identify isoform-specific ESTs or paralogues (Table 3). The expression pattern of a number of these ESTs, including the complete set of 40S and 60S ribosomal proteins, has been further characterized during spermatogenesis and larval development [29-32,40,41].

**Table 3:** Examples of some ESTs encoding mRNAs of potential interest during gametogenesis and development in Senegalese sole.

GenBank acc. no	Gene identification (species) of top BLAST hit^a^	GenBank hit acc. No	BLAST E-value	Length (% identity)^b^
FF291770	Steroidogenic acute regulatory protein (*Equus caballus*)	O46689	2E-25	55 (50%)
FF285271	17-beta hydroxysteroid dehydrogenase type 12B (*Danio rerio*)	Q6QA33	6E-42	87 (72%)
FF288093	Inhibin alpha subunit (*Fundulus heteroclitus*)	AAW02847	9E-43	76 (73%)
FF282090	Cyclin B1 (*Orizyas latipes*)	Q9IBG1	1E-29	66 (81%)
FF287053	Cyclin B2 (*Oncorhynchus mykiss*)	Q09IZ1	3E-43	93 (72%)
FF287848	Cyclin D1 (*Danio rerio*)	Q90459	3E-32	89 (78%)
FF284385	Gonadotropin I beta subunit (*Paralichthys olivaceus*)	AAK58601	4E-50	89 (74%)
FF285638	Gonadotropin II beta subunit (*Morone saxatilis*)	AAC38019	9E-35	64 (81%)
FF291056	Gonadotropin alpha subunit precursor (*Epinephelus coioides*)	AAN18039	1E-48	86 (86%)
FF283815	Forkhead box F2 (*Xenopus tropicalis*)	A4IIG1	2E-75	156 (64%)
FF282163	Transcription factor jun-B (*Cyprinus carpio*)	AAB39939	6E-28	65 (92%)
FF283387	Testis-specific gene A2-like protein (*Cyprinus carpio*)	Q6VTH5	3E-10	24 (46%)
FF290593	Creatine kinase testis-isozyme (*Oncorhynchus mykiss*)	P24722	6E-84	162 (90%)
FF290243	Vitellogenin C (*Morone americana*)	AAZ17417	4E-40	90 (66%)
FF283702	Apolipoprotein A-I (*Morone saxatilis*)	ACH90229	2E-25	48 (53%)
FF285567	Apolipoprotein A-IV1 (*Takifugu rubripes*)	Q5KSU4	8E-70	139 (61%)
FF283106	Apolipoprotein A-IV4 (*Takifugu rubripes*)	Q5KSU1	5E-35	73 (64%)
FF282216	Elongation factor 1 alpha (*Oryzias latipes*)	Q9YIC0	3E-35	76 (93%)
FF287281	Elongation factor 1 beta (*Oryzias latipes*)	CAB40840	3E-32	67 (90%)
FF287587	Elongation factor 1 gamma (*Danio rerio*)	Q8JIU6	9E-90	148 (84%)
FF285240	Elongation factor-1, delta isoform 1 (*Danio rerio*)	B0S5L3	6E-61	122 (78%)
FF282440	Myosin, heavy polypeptide 1, skeletal muscle (*Danio rerio*)	Q7T1B7	1E-124	249 (90%)
FF291822	Myosin 10 (*Xenopus laevis*)	Q694W8	6E-32	65 (83%)
FF287487	Myosin, heavy polypeptide 11, smooth muscle (*Danio rerio*)	Q4U0S2	1E-94	217 (92%)
FF289234	Heat shock protein 90-alpha (*Danio rerio*)	Q90474	3E-121	225 (95%)
FF291891	Heat shock protein 90 beta (*Paralichthys olivaceus*)	AAQ95586	3E-57	163 (91%)
FF288187	Heat shock protein 70 (*Poecilia reticulata*)	ABN80448	2E-20	51 (68%)
FF281871	Keratin type I, CK18 (*Danio rerio*)	Q7ZTS4	2E-104	195 (84%)
FF283334	Keratin type I, CK19 (*Pongo abelli*)	Q5R8S9	3E-53	104 (70%)

^a^Most significant BLASTX hit is reported; ^b^Extent of BLASTX hit aligned region (in amino acids), and percent identity over the aligned region.

### Design and validation of a Senegalese sole oligo microarray

We developed an oligonucleotide microarray for studies on *S. senegalensis *gene expression given the greater reproducibility of the data collected with oligonucleotide as opposed to spotted cDNA microarrays [42]. *Tethys*, the proprietary oligo design software from Oryzon Genomics, was used to design an oligo microarray based on the ESTs sequenced. As mRNA labelling procedures such as the Eberwein protocol [43] are biased towards the 3' polyA tail of the transcripts, oligo design was equally biased towards the 3' end of the sequences. Specific 50 to 60-mer oligonucleotide probes were successfully designed for 5,087 out of the 5,208 unigenes assembled from the ESTs (Additional file 1). We also designed probes for control mRNAs, which were spiked into each mixture prior to hybridization, for quality control monitoring and data processing. The final design of each slide, comprising 2 arrays with gene specific probes for all 5,087 *S. senegalensis *unigenes as well as negative and positive control oligos, was submitted for synthesis to Agilent using the eArray platform.

**Figure 3:**
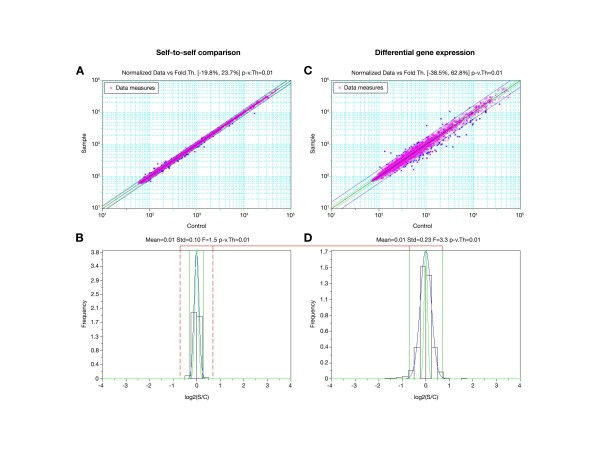
**Senegalese sole microarray platform**. Scatter plot of the signal intensities in the Cy3 and Cy5 channel of replica analysis of self-to-self (A) and differential gene expression (C) experiments. In both panels, the green and blue lines parallel to the black diagonal line represent the ± 3σ limits of the data from control and *S. senegalensis *specific oligos, respectively. The histograms of the distribution of fold changes (FC) as log2(S/C) for control and *S. senegalensis *specific oligos in the self-to-self (B) and differential gene expression (D) experiments are also shown. In these panels, the green and blue curves represent the ± 3σ limits on the data from control and *S. senegalensis *specific oligos, respectively, and the red dotted lines in the self-to-self histogram represent the cut-off chosen in the differential gene expression experiment to estimate overall FDR.

Two independent experiments were conducted to estimate the rate of false-positive expression in the microarray. In the first experiment, self-to-self hybridization, total RNA from two different aliquots of the same sample of sole ovary were used to produce either Cy3 or Cy5 differentially labelled amplified RNA (aRNA). The aRNAs were mixed in equal amounts, with the fluorescent cyanine dyes Cy3 and Cy5, and hybridized in triplicate to the microarray (Figure 3A and 3B). In the second experiment, mRNA from sole larvae was used, from 16-day post-hatching to produce Cy3 labelled aRNA and from 22-day post-hatching to produce Cy5 labelled aRNA, mixed in equal amounts and hybridized in triplicate to the sole microarray (Figure 3C and 3D). We assessed whether the variation of all three fold change (FC) values were within the limits of expected variation, and any considered as an outlier (e.g., due to imperfections in the array), were eliminated. From this analysis we calculated the mean FC value for each oligo on the array and the distribution (Figure 3B and 3D).

As expected, the narrow distribution obtained with the identical RNA sources in self-to-self hybridization indicated very few differences in gene expression (Figure 3B). In contrast, a much broader distribution was observed for the gene specific probes in the differential gene expression experiment (Figure 3C and 3D), with distribution of the control oligos similar to that in the self-to-self experiment. Individual error rates are a function of the FC and the reproducibility of the values for a single oligo in the experiment, but comparison of the distribution of the gene specific data between self-to-self and differential gene expression experiments gives an estimation of the overall false discovery rate (FDR). The FDR of a test is defined as the expected proportion of false positives in the significant results, and can be estimated by dividing the number of data points or surface under the data curve of the self-to-self (or control) hybridization in the histogram beyond a chosen FC cut-off (e.g., Figure 3B), by the datapoints or surface beyond the same FC cut-off under the data curve from the differential gene expression (e.g., Figure 3D). Overall, the FDR is expected to increase with lower cut-off FC values, and decrease when the dispersion of the self-to-self data is low, i.e. in function of the overall reproducibility of the microarray platform. In the differential gene expression experiment shown in Figure 3C, we identified 21 up-regulated genes and 71 down-regulated genes, with a *p-value *≤ 0.01 for genes considered significantly expressed and an FC cut-off ± 3σ, in this case equal to ± 1.62. In the same interval, we identified 1 up-regulated and 2 down-regulated genes for the self-to-self experiment, giving an estimated overall FDR of 3.26%. These results indicate excellent technical reliability of the platform.

The microarray developed may be of great interest in research on Senegalese sole gene expression, given the range of questions that it can be used to address. This array, has already been employed within the Pleurogene program to investigate changes in the transcriptome profiling during gonad differentiation, growth and maturation, and during larval metamorphosis and development. Additional microarrays can be obtained from Agilent using the EST-specific oligos listed in the Additional file 1.

### Development of the Soleamold bioinformatic platform

**Figure 4:**
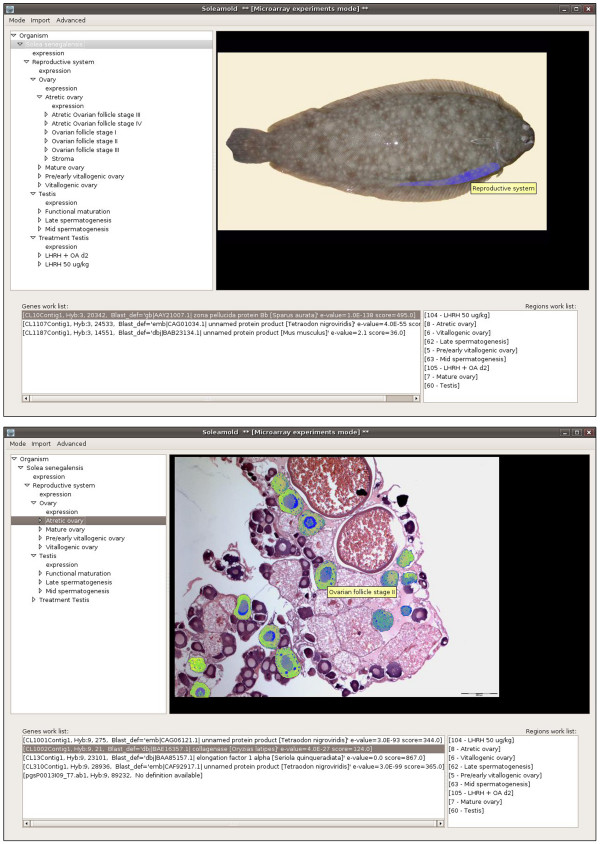
**Soleamold main interface and navigation of the atlas**. The upper panel shows the Soleamold main interface which is divided into three interrelated sections: a hierarchical tree with the ontology describing the organism (upper left), an atlas coherent with the terms of the ontology (upper right), and two work lists for the regions (i.e., samples) and genes of interest. All parts of the interface are interactive and/or navigable through contextual menus and have been designed to be visually intuitive, allowing for constant contextualization of the data. The lower panel shows that the construction of an atlas coherent with the ontology allows for the construction of maps that, when overlaid with the original picture, highlight the different regions that can be identified in the picture. Thus, users can slide the cursor over any image and rapidly identify and/or select each region in it. In the figure, follicles at stage II from an atretic ovary are highlighted.

The use of functional genomics approaches can be highly implemented towards the discovery of new genes and genetic pathways by determination of the cellular localization of gene expression using ISH. However, most current databases that integrate both approaches on an anatomical basis in vertebrates are based on model organisms, such as mouse [44] or zebrafish [45] embryos. The Soleamold platform was developed as a bioinformatic resource able to manage and store all experimental data on gene expression levels and gene localization in *Solea senegalensis *[46]. This platform is based on the Orymold software [47] which integrates microarray experimental data through an ontological description of organs, tissues and cell types of a given organism hierarchically. The current ontology of Soleamold describes *Solea senegalensis *male and female reproductive organs using a total of 94 terms, from 'ovary' and 'testes' to 'late spermatogenesis' and 'follicle maturation stages', but can be extended to cover many different organs and experimental treatments (Figure 4). The ontology is complemented with histological images, forming a coherent, user-navigable atlas that backs up the meaning of the terms included in the hierarchical description of Senegalese sole. The user can input new data to both atlas and ontology.

**Figure 5:**
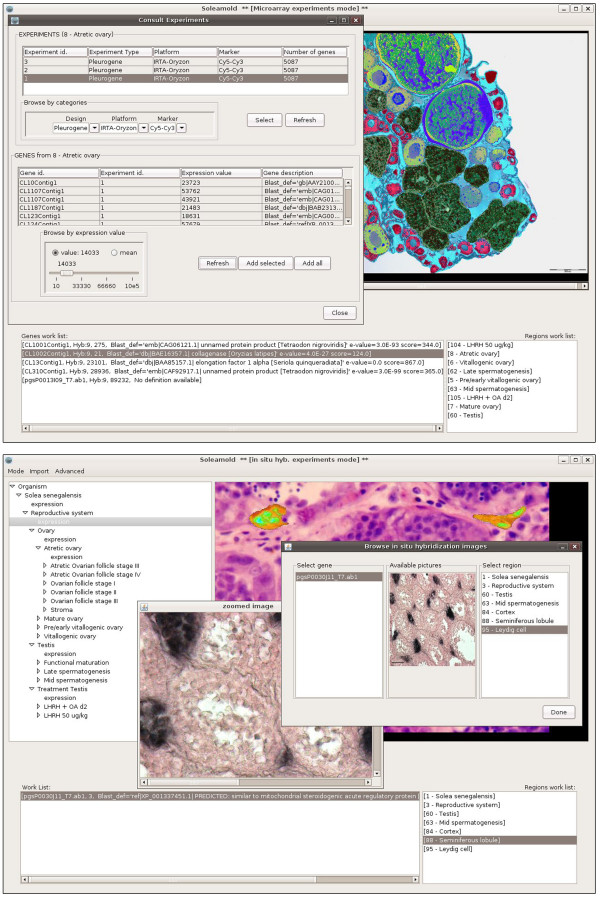
**DNA Microarray and ISH modules**. By consulting a term of the ontology a pop-up frame allows the user to browse DNA microarray experiments present in the database, as shown in the upper panel. Experiments can be browsed using several parameters: chip design, hybridization platform and labelling. Once an experiment is selected, those genes showing expression appear in the lower list. Expression values can then be selected according to a lower threshold value and sent to the work list of the main interface for further consultation. The lower panel shows that Soleamold also allows for the storage, consultation and interpretation of ISH experiments. The figure shows the distinct browsing interfaces for this type of data. In the foreground, the original experimental pictures browsing interface shows steroidogenic acute regulatory (StAR) mRNA expression in Leydig cells, and its zoomed image. In the background, there is the automatically built, atlas image, of the same experiment showing the expression in Leydig cells as interpreted by the user at the insertion time.

The Soleamold platform integrates experimental data from DNA microarrays and ISH based on the ontology (Figure 5). Once integrated, data can be easily browsed and retrieved. The genome of *Solea senegalensis *has not been sequenced yet, and therefore it was not possible to build a genomic knowledge database to serve as backend support for experimental data. Instead, a BLAST-able [48] database derived from the clustered and annotated sole EST collection was incorporated into the tool. This database dynamically maps and annotates each of the probes contained in a DNA microarray or employed for ISH. Once probes have been introduced into the system, experimental data is inserted by selecting a term in the ontology and uploading the expression values that are automatically linked to the backend database through probe names. Users are also able to determine the quality of the introduced data and comparability of the results. When dealing with microarray data, the same reference sample must be used for all hybridization experiments, ensuring future cross-comparability of data.

The information in Soleamold is retrieved while maintaining constant visual contact with the ontology and the atlas, demonstrating the biological significance of the gene expression data. Users can make semantic queries, such as 'Where or when do gene X and gene Y show differential expression, so that gene X is overrepresented while gene Y has a low level of expression?', based on ontology terms that allow for the isolation of genes whose expression values meet specific criteria spatially and temporally. Semantic queries allow for the construction of lists of elements of interest based on the model ontology, therefore checking and validating both the experimental data and the model.

The current experimental database of Soleamold includes nineteen normalized and scaled microarray hybridizations to compare the global pattern of gene expression during ovarian and testicular development, including data from males treated with different hormones, and few ISH experiments. Detailed analysis of these experiments, as well as validation of the FC variations in expression by real-time quantitative PCR, will be published elsewhere. The Solemold can be extended to additional tissues and organs of Senegalese sole, as well as of other flatfish, after normalization of microarray gene expression data.

## Conclusion

The lack of genomic resources for non-model fish species of commercial interest has been in stark contrast to the increasing importance of aquaculture to counteract the world-wide decline in wild fishery. By sequencing and analyzing 10,185 *Solea senegalensis *ESTs, we have generated new genomic resources for this commercially important flatfish. In addition to contributing to the characterization of fish genes of unknown function, the EST survey will be useful to identify molecular markers for future genetic studies in Senegalese sole and to provide probes for ISH cellular localization studies. The DNA microarray can be used for further investigation of physiologically and ecologically relevant transcriptional regulation in *Solea senegalensis*. Finally, the Soleamold interactive morphoanatomical atlas of gene expression may aid in the identification of crucial genes and regulatory pathways involved in different physiological process in flatfish, as such, it may be a powerful research tool for both academia and industry.

## Methods

### Animals and sampling

Adult, sexually mature Senegalese sole males and females (*n *= 25; 749 ± 36 g in weight) were captured in the salt marshes of the bay of Cadiz (Spain). Fish were maintained in 28 m^2 ^round fibre glass tanks in the laboratory for approximately 30 days under natural conditions of photoperiod and temperature as described [49]. After this period, fish were killed by sedation in 100 ppm 2-phenoxyethanol and decapitation, and samples of the gonads, liver, brain (including pituitary), stomach and gut were rapidly dissected. Larvae were raised from fertilized eggs spawned by wild-caught broodstock adapted to captivity and maintained under ambient conditions as described [40]. Samples of larvae were collected before metamorphosis (up to day 12 postfertilization; 6–7 mm) and during and after metamorphosis (from day 14 up to day 23 postfertilization; 8–9 mm). Samples of larvae with albinism, malpigmentation, osteological malformations, and incomplete eye migration, were also taken. Samples from undifferentiated gonads were taken from approximately 120 fish between 138 and 205 days post-fertilization (43–112 mm total length). In all cases, tissues were flash-frozen in liquid nitrogen and stored at -80°C prior to RNA extraction. The protocols for the sacrifice of fish were approved by the Ethics and Animal Experimentation Committee from IRTA (Spain) in accordance with the Guiding Principles for the Care and Use of Laboratory Animals.

### cDNA libraries and normalization

Total RNA was extracted from frozen adult tissues (from 3–5 different fish) and larvae using the RNeasy kit (Qiagen), and poly(A)^+ ^RNA purified using the Oligotex Direct mRNA Mini Kit (Qiagen). For larvae, samples at the same developmental stage were pooled to obtain 1 g total tissue for RNA extraction. Ten different cDNA libraries (Table 1) were unidirectionally constructed (5' *Eco*RI, 3' *Xho*I) using the ZAP Express^® ^cDNA Synthesis Kit following the manufacturer's instructions (Stratagene). Analysis of size-fractionated *Xho*I-digested cDNAs, performed immediately prior to ligation into the vector, was done using 5% non-denaturing acrylamide gel electrophoresis or the 2100 Bioanalyzer (Agilent) and DNA 7500 LabChip kit (Agilent). For normalization, an aliquot of each library, depending on its titre after one round of amplification (Table 1), was pooled into a master library which was mass-excised according to the manufacturer's instructions, and subsequently normalized through three successive rounds of plating and hybridization. In the first round, colonies were plated on large LB^+ ^kanamycin plates and 384 clones selected randomly and transferred to 384-well plates. A ^32^P labelled probe was prepared from the inserts of the pool of these selected clones using PCR and primers corresponding to either end of the multicloning site of the vector. For the second hybridization round, we plated the library and obtained colony blots on Hybond N^+ ^filters (Amersham), following the manufacturer's instructions. These were hybridized with the first PCR probe. From the second round of screening, 3,456 non-hybridizing clones were selected and transferred to 384-well plates. The pool of these selected clones was then used to prepare a second radioactive labelled PCR probe which was hybridized to additional colony blots. From this third round of normalization, 6,528 non-hybridizing colonies and all selected clones were sequenced.

### Sequencing, sequence analysis and contig assembly

The normalized master library (10,368 cDNA clones) was arrayed in Genetix X7001 flat 384-well plates. Glycerol stocks of overnight cultures were prepared in 384-well format. Plasmid DNAs were extracted from cultures grown overnight in deep 384-well plates containing 200 μl of 2YT Media. Plasmids were isolated and purified automatically by a modified alkaline lysis procedure. Quality control of plasmids was done by randomly testing eight samples of each plate on agarose gels. Sequences were determined using the T7 universal primer, ABI Big Dye Terminator 3 chemistry and ABI 3730XL capillary sequencer systems (ABI, Weiterstadt, Germany). Base-calling from chromatogram traces was performed using PHRED [50,51]. Vector, *Escherichia coli *contamination, and low-quality regions were trimmed from EST sequences.

Before clustering, EST sequences were subjected to an extensive quality control procedure. Firstly, sequences were clipped at both ends as long as PHRED quality values stayed below 20 in a window of 15 bp in size. Secondly, putative vector and repeat sequences were masked for all sequences. PHRAP [52], under stringent clustering parameters (minimum score, 100; repeat stringency, 0.99), was used to assemble ESTs into contigs. Consed (Version 14.00) [53] was used for final editing of the sequence. Contig consensus sequences and singleton sequences were aligned with nonredundant GenBank nucleotide and amino acid sequence databases using BLASTN and BLASTX, respectively [48,54].

### Functional characterization of ESTs

Senegalese sole contig consensus sequences and singleton sequences were submitted for GO annotation to the online version of the BLAST2GO v1 program [55,56]. Annotated accession numbers and GO numbers were derived with NCBI's QBLAST, with an expectation E-value ≤ 10^-3 ^and an HSP length cut-off of 33. Contig sequences were then annotated according to the following parameters: a pre-E-value-Hit-Filter of 10^-6^, a pro-Similarity-Hit-Filter of 15, an annotation cut-off of 55, and a GO weight of 5. Directed acyclic graphs (DAG's) were generated using a sequence filter of 5, an alpha score of 0.6 and a 0 node score filter. A score was computed by Blast2GO to highlight areas of high annotation. This score was computed at each node according to the following equation:

$$\text{Score}=\sum_{GOs}seq\times \alpha^{dist}$$

where *seq *is the number of different sequences annotated at a child GO term, *dist *the distance to node of the child GO term, and α the parameter entered by the user. The data presented represent the level 2 analysis, illustrating general functional categories.

### Oligo design, microarray fabrication and quality control

The EST unigenes were used to design microarray oligoprobes using *Tethys*, Oryzon's proprietary software which is based on the thermodynamic simulation of hybridization *in silico*. The final array design contained 5,087 gene specific *Solea senegalensis *probes, as well as positive and negative control probes (maize expansin, ZmMYB42, and xyloglucan endo-transglycosylase), a total of 700 per array. These were used to assess detection limits and range, to verify spatial homogeneity, and to determine experimental within-array variation. Microarray slides, each containing two microarrays, were synthesized by Agilent using ink-jet printing and *in situ *oligonucleotide synthesis.

### RNA extraction, microarray hybridization and analysis

Total RNA was extracted from Senegalese sole ovaries and testes using the RNeasy extraction kit (Qiagen) followed by DNAse treatment. The quality and concentration of the RNA was analyzed using the Agilent 2100 bioanalyzer and NanoDrop™ ND-1000 (Thermo Scientific). Samples with RNA integrity number (RIN; [57]) < 6.0 were discarded. Total RNA (0.5 μg) amplification and labelling with Cy3 or Cy5 was carried out using the Eberwein mRNA amplification procedure [43] employing the MessageAmp™ aRNA amplification kit from Ambion (Applied Biosystems) following the manufacturer's instructions with minor modifications.

The Cy3- and Cy5-labelled cRNAs were combined and hybridized to the microarray for 17 h at 60°C using Agilent's gaskets G2534-60002, G2534A hybridization chambers and DNA Hybridization Oven G2545A, according to the manufacturer's instructions. Raw data were obtained using Agilent's DNA Microarray Scanner G2505B and Feature Extraction software (v10.1). The raw fluorescence intensity data were processed using the *Polyphemus*™ software, developed at Oryzon Genomics, which includes spatial data compensation, non-significant expressed data filtering, and data normalization. Data normalization was carried out by an improved version of the nonlinear *Q-splines *normalization method [58], enhanced with robust regression techniques. Normalized and log-transformed data were used to calculate FC values. Differential expression was assessed with *Polyphemus*™ analysing biological replicates based on repeated experiments using robust statistics on average technical replicates removing the outlier points (caused by dust or array imperfection). The *p*-values were calculated based on the absolute value of the *regularized t-statistic *[59], which uses a Bayesian framework to derive the algorithm, using internal replicated controls to assess the minimum technical variability of the process. The *regularized t *approach is more sensitive than the significance analysis of microarrays (SAM) method [60,61]. Cut-offs for significant changes were chosen in various ways, but they were always greater than the inherent experimental variation as assessed by the FC of internal controls and/or self-to-self hybridizations.

### Histology and ISH

Samples of gonads were transferred into Bouin fixative for approximately 48 h at room temperature, and subsequently dehydrated and embedded in Paraplast (Sigma). In situ hybridization of eosin-haematoxylin stained sections (7 μm) was carried out with digoxigenin-alkaline phosphatase (DIG-AP) incorporated cRNA probes as described [41]. DIG-AP riboprobes were synthesized with T3 and T7 RNA polymerases using the DIG RNA Labeling Kit (Roche). Sections were examined and photographed with a Leica DMLB light microscope.

### Construction of Soleamold

Soleamold was designed following a two-tier architecture: a desktop standalone client in charge of data presentation and processing, and a relational database management system (DBMS) for handling data organization, storage and retrieval. This architecture allows for concurrent remote access by users in a fairly scalable manner. Standalone applications are more difficult to implement but they allow a higher degree of interactivity, and in general, for a much richer user experience than those that are web based. Java J2SDK [62] was chosen for client implementation to ensure portability through operative systems while MySQL [63] was chosen as DBMS. MySQL proprietary Java Database Connectivity (JDBC) driver was used for client connection. Soleamold is available through the Spanish National Institute of Bioinformatics website [44].

## Abbreviations

EST: Expression sequence tag; ISH: In situ hybridization; ZP: Zona pellucida; GO: Gene ontology; FDR: False discovery rate; SAM: Significance analysis of microarrays; DMS: Database management system.

## Authors' contributions

JC conceived and designed the study. MM, CI, AA y GMR constructed the larvae cDNA libraries, JV and FP the undifferentiated gonads library, SH, FP and GMR the brain library, CI, AA, MY and GMR the stomach, intestine and liver libraries, ATS and JC the ovarian library, and BC and JVP the testis library. JC and JJL performed the bioinformatic analyses of ESTs. OD, FS and ER, assisted by TM, normalized the sole multi-tissue cDNA libray, designed microarray oligonucleotides and carried out microarray hybridizations and bioinformatic analyses. JM and TM, assisted by JC and ATS, designed and constructed the Soleamold. JC and TM wrote and edited the manuscript. All authors read and approved the manuscript.

## Supplementary Material

Additional file 1 **Sequence of oligos for each *S. senegalensis *unigene employed in the microarray.** Click here for file
